# Meniscal Injuries in Patients Aged 40 Years or Older: A Comparative Study Between Meniscal Repair and Partial Meniscectomy

**DOI:** 10.7759/cureus.33270

**Published:** 2023-01-02

**Authors:** Moisés Ventura, Pedro Seabra, José Oliveira, Paula Sousa, Miguel Quesado, Henrique Sousa, Ricardo Pereira, André Costa, Paulo Carvalho

**Affiliations:** 1 Orthopaedics and Traumatology, Centro Hospitalar Vila Nova de Gaia/Espinho, Vila Nova de Gaia, PRT

**Keywords:** knee, arthroscopy, trauma, age, meniscectomy, meniscal repair

## Abstract

Introduction

Meniscal tears represent one of the most frequent knee injuries and are the most common cause of knee surgery. Historically, age has been considered an independent factor contraindicating meniscal repair due to the assumption that meniscal injuries in this population are frequently chronic tears, mostly with a degenerative tear pattern, and low healing potential. However, recent literature has questioned this paradigm with studies reporting successful outcomes with meniscal repair in older patients. Our study aimed to evaluate and compare the short-term clinical outcomes of meniscal repair versus partial meniscectomy in patients aged ≥40 years old.

Methods

A retrospective study was conducted that included patients over the age of 40 years, diagnosed with meniscal tears, that underwent arthroscopically assisted meniscal repair or partial meniscectomy between 01 January and 31 December 2020. The patients were divided into two groups: Group 1- partial meniscectomy (PM) and Group 2- meniscal repair (MR). The clinical evaluation was performed 24 months after the surgery, and the studied variables were: function (Tegner Lysholm Knee Scoring Scale), pain (Visual Analogue Scale), patient satisfaction, and failure rate.

Results

Fifty-one patients met the inclusion criteria, and 7 were excluded due to loss of follow-up during telephone contact. Thus, the final sample consisted of 44 patients (mean age 52.18y), both groups with 22 patients. In both groups, we found an improvement in pain 2 years after the surgery, with a decrease in the VAS value between the pre and post-surgery. On average, the VAS score decreased from 7.9 to 4.5 in the group subjected to partial meniscectomy, and from 7.5 to 3.2 in the meniscal repair. This was statistically significant in both groups, with a p-value <0.01, but not between them (p-value = 0.363). Comparing the degree of satisfaction between both groups, we found no statistically significant difference between them (p=0.167). Regarding the functional outcome (Tegner Lysholm Knee Scoring Scale), the group that underwent the meniscal repair obtained a statistically superior score compared to the partial meniscectomy group (77.55 vs. 64.77; p-value 0.033). The failure rate was exactly equal in both groups (4.5%), therefore no statistically significant difference was found in this variable.

Conclusion

Age, as an independent factor, should not be considered a contraindication for meniscus repair. In fact, if technically possible, meniscal repair should always be performed as it is associated with better functional outcomes, similar failure rates, and may be protective against the development and progression of arthritis.

## Introduction

Meniscal tears represent one of the most frequent knee injuries and commonly need surgical treatment due to pain or mechanical symptoms. Historically, menisci were considered vestigial remnants and were frequently resected [1]. Fairbank in 1948 suggested that meniscectomy predisposed premature degenerative changes in the knee joint, and described the radiographic changes that followed this procedure and culminated in knee osteoarthritis. Subsequently, several studies confirmed the poor long-term function and early degenerative changes in patients post-meniscectomy [2]. Recently, various important roles like contribution to load sharing, shock absorption, joint stabilization, proprioception, and lubrication of the joint have been attributed to the menisci [3]. 

Population studies demonstrated that meniscal tears requiring treatment are two to three times more common in patients over 40 years than in those younger [4,5]. Recent evidence has questioned the benefit of meniscectomy in this patient population [6-8]. Total meniscectomy reduces the contact area by 75%, increases the peak local contact stresses by 235%, and allows for increased anterior translation of the femoral condyle on the tibia [9]. These increased shear forces and compressive loads are believed to result in arthritic changes [2,10,11]. Partial meniscectomy may produce the same change, with variability based on the amount and location of meniscal resection. For that reason, meniscus repair has emerged as a possible treatment for meniscal injuries in this age group [12].

However, the expected outcomes of repair in this population are ambiguous. Historically, age has been considered an independent patient-related contraindication for meniscal repair due to the assumption that meniscal injuries in this population are frequently chronic tears, mostly with a degenerative tear pattern (horizontal cleavage), and are associated with degenerative cartilage changes [1, 13-15]. In a study conducted by Mesiha et al., the histological characteristics of 44 meniscal tears were reviewed and the authors found decreased intrinsic and perimeniscal cellularity in patients ≥40 years old compared with the control group [16]. Menisci with few or no intrinsic cells are more disposed to acute or degenerative tears, while the presence of viable “normal” meniscal cells is a significant factor for meniscal survival [1] This may contribute to a lower healing potential, and constitutes an argument against meniscal repair in this age group.

Despite this, some studies reported successful results for meniscal repair in an older population [12,17-19]. Barrett et al. [12], conducted a study with 37 patients and obtained a high clinical success rate (86.5%) at 26.5 months in patients aged on average 44 years. Only 5 patients had a recurrence of symptoms in which further arthroscopies were needed. Noyes et al. [17] evaluated the repair outcomes in a population with a mean age of 45 years who underwent meniscal repair with or without anterior cruciate ligament (ACL) reconstruction and obtained very good/good outcomes in 88% of patients, with only 3 requiring a meniscectomy at 33-month follow-up. These authors recommended that meniscal repair should be considered in active patients, regardless the age [17].

The purpose of this study is to evaluate and compare the short-term clinical outcomes of meniscal repair versus partial meniscectomy in patients aged ≥40 years old.

## Materials and methods

Patient selection, operative technique, and postoperative care

We conducted a retrospective study including patients over 40 years diagnosed with meniscal tears that underwent arthroscopically assisted meniscal repair or partial meniscectomy between 01 January and 31 December 2020. Anterior and posterior root avulsion injuries were excluded from this study.

The following medical data were collected: sex, age, laterality, sports participation, injury location, rupture type, associated lesions (presence of cartilage and ligament injury), type of treatment (partial meniscectomy or meniscal repair), concomitant procedures, complications, and reoperations. Twenty-four months after surgery, patients were contacted by telephone to obtain clinical and functional results. These were assessed using the Tegner Lysholm Knee Scoring Scale (LKSS), the degree of satisfaction with the surgery was scored from 1 to 5, and the degree of pain was evaluated in two moments: before the surgery and 24 months after the surgery, using the visual analogue scale (VAS).

The patients were divided into two groups, Group 1: patients that underwent partial meniscectomy (PM), and Group 2: patients that underwent meniscal repair (MR). The decision for meniscal repair versus partial meniscectomy took into account the surgeon’s assessment of tissue quality, tear pattern, tear vascularity, and surrounding cartilage status.

Meniscal tears were defined by pattern (radial, vertical or longitudinal, horizontal, or complex pattern). During the arthroscopy, cartilage injuries were recorded according to the Outerbridge classification and were treated with either debridement, chondroplasty, or microfracture. Anterior cruciate ligament (ACL) status was also evaluated and in case of injury, ACL reconstruction was performed with concomitant meniscectomy or meniscal repair.

Repair techniques used for the treatment of the meniscal tears in group 2 consisted of all-inside, outside-in, and inside-out techniques, depending on the location of the tear and the surgeon’s choice. This variable was not specified for each patient because we considered it beyond the scope of this article. All-inside repairs were performed with Fast-Fix 360® implants (Smith & Nephew), outside-in and inside-out with 2-0 braided sutures (Smith & Nephew). As an adjunct procedure, rasping of the meniscus, trephination, or bone marrow venting was performed at the time of the repair to stimulate healing. In 2 cases of meniscal repair, an additional meniscal centralization technique was performed in which the tibial fixation was performed with an anchor (1.4 mm JuggerKnot® Soft Anchor System - Zimmer Biomet). Additionally, a concomitant ACL reconstruction was performed in 1 patient of group 1 (PM) and 2 patients of group 2 (MR).

After the meniscal repair, the patients were kept non-weight bearing for 4-6 weeks, allowing restricted knee mobilization (from full extension to 90º of flexion) for 6 weeks. The group that underwent partial meniscectomy was allowed to perform a full range of motion of the knee and was advised to use crutches for 2 weeks for partial weight bearing and to progress gradually to full weight bearing as tolerated. All patients were referred for physical therapy.

Outcomes measures

Functional outcomes were measured using the Tegner Lysholm Knee Scoring Scale (LKSS), a patient-reported instrument that consists of 8 different items (pain, instability, locking, swelling, limp, stair climbing, squatting, and the need for support) on a 100-point scale - range from 0 (worse disability) to 100 (less disability) [20].

The visual analogue scale (VAS), range 0-10, was used to classify the amount of knee pain during daily life activities. It was measured at two time points: before the surgery and at the end of the follow-up (24 months after surgery). In addition, the difference between the value obtained before the surgery and 2 years after the surgery was also calculated.

Regarding the degree of satisfaction with the surgery, it was measured using a linear scale from 1 to 5 (1 corresponding to “Very Dissatisfied” and 5 to “Very Satisfied”).

Failure was defined as subsequent surgery: revision of the meniscal repair/meniscectomy or knee arthroplasty (total or unicondylar), performed on the original compartment of the surgically repaired meniscus. Meniscal surgery on the contralateral meniscus was not considered a failure.

Statistical analysis

Statistical analyses were conducted using a standard software package IBM SPSS v21. Descriptive statistics were generated for the entire data set. Nominal variables were tested with the Chi-square test. Independent Samples t-Test was used to analyze independent numerical variables, while Paired Samples t-Test was used for dependent numerical variables. We used a significance level of 0.05.

## Results

Fifty-one patients met the inclusion criteria while 7 were excluded due to loss of follow-up during telephone contact. Thus, the final sample consisted of 44 patients (mean age 52.18y), both groups with 22 patients each.

The group that underwent partial meniscectomy (PM) had a mean age of 53.41 years, while the group of meniscal repair (MR) had a mean age of 50.95 years, with no statistical differences between them (p=0.307).

The demographic variables are presented in table 1.

**Table 1:** Descriptive Statistics

Demographics	Partial Meniscectomy	Meniscal Repair	Total	p-value
Age [years-old] - mean (standard deviation)	53.41 (7.53)	50.95 (8.21)	52.18 (7.89)	0.307
Laterality - frequency				0.227
Right	8	13	21	
Left	14	9	23	
Sex - frequency				0.364
Male	10	14	24	
Female	12	8	20	
Physical Activity - frequency				0.346
Yes	13	13	26	
No	9	9	18	
Meniscus - frequency				0.709
Medial	18	17	35	
Lateral	4	5	9	
Location of the meniscal tear - frequency				0.460
Anterior horn	1	0	1	
Posterior horn	6	6	12	
Body	9	5	14	
Body + Anterior horn	1	2	3	
Body + Posterior horn	5	9	14	
Type of tear - frequency				0.521
Bucket handle	2	2	4	
Longitudinal	1	2	3	
Horizontal	10	5	15	
Radial	1	3	4	
Complex	8	10	18	
ACL injury with reconstruction - frequency	1	2	3	0.55
Cartilage damage – Outerbridge Classification - frequency				0.646
0	5	8	13	
1	1	1	2	
2	7	7	14	
3	2	3	5	
4	7	3	10	

**Figure 1:**
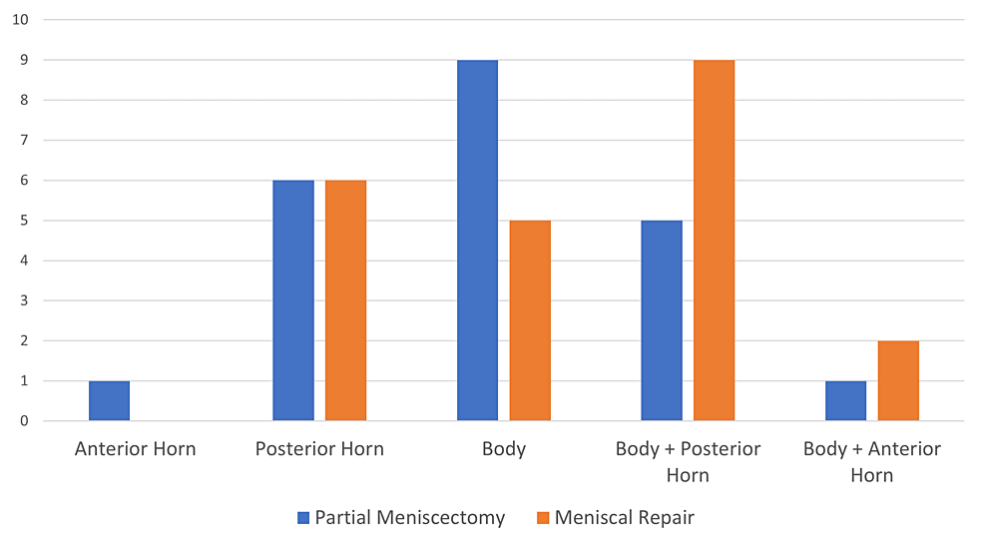
Distribution of the meniscal tears acording to the location on the meniscus and type of treatment (frequency)

We found a slight predominance on the left side (52.3% on the left vs. 47.7% on the right). The medial meniscus was the most prevalent (79.5%). Of the 35 medial menisci, 17 were subjected to meniscal repair and 18 to partial meniscectomy. As for the 9 lateral menisci, 5 underwent meniscal repair while 4 underwent a partial meniscectomy. Most injuries were located in the body and/or posterior horn of the meniscus (90.9%) (Figure 1).

**Figure 2:**
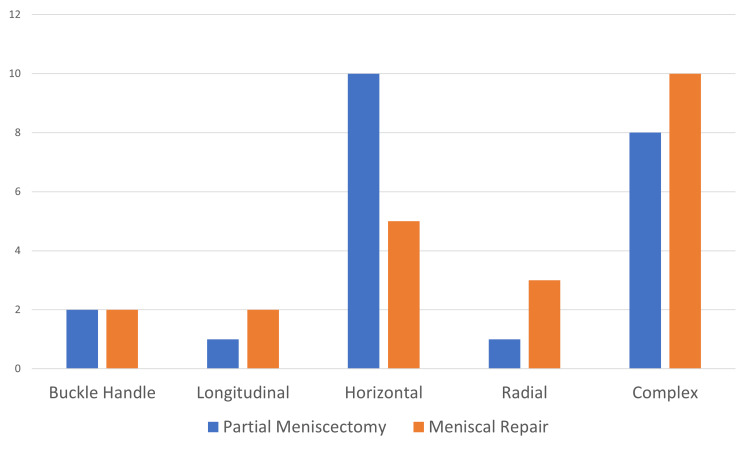
Distribution of the pattern tears according to the type of treatment (frequency)

Regarding the type of tear, we identified a predominance of horizontal (34%) and complex (41%) patterns in both groups (Figure 2).

Clinical outcomes

**Figure 3:**
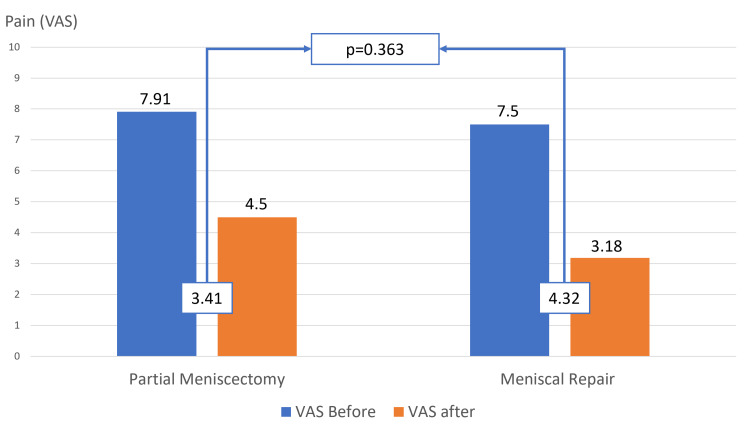
Pain score before and 24 months after the surgical treatment

In both groups, we found an improvement in pain 2 years after the surgery, with a decrease in the VAS value between the pre and post-surgery. On average, the VAS score decreased from 7.9 to 4.5 in the group subjected to partial meniscectomy, and from 7.5 to 3.2 in the meniscal repair. This was statistically significant in both groups, with a p-value <0.01 (Figure 3).

When calculating the difference in the mean VAS score before and after the surgery, we found a slight improvement in the group that underwent meniscal repair. However, this difference was not statistically significant (PM:3.41 vs MR 4.32; p-value = 0.363) (Table 2).

**Table 2:** Clinical and Functional outcomes

Outcomes	Partial Meniscectomy	Meniscal Repair	Total	p-value
Pain Before (VAS) – mean (standard deviation)	7.91 (1.74)	7.5 (1.37)	7.7 (1.56)	0.392
Pain After (VAS) – mean (standard deviation)	4.5 (3.17)	3.18 (2.87)	3.84 (3.06)	0.156
Difference of pain (Pain before-Pain After) – mean (standard deviation)	3.41 (3.81)	4.32 (2.64)	3.86 (3.27)	0.363
Tegner Lysholm Knee Scoring Scale – mean (standard deviation)	60.7 (30.6)	77.5 (18.1)	69.11 (26.26)	0.033
Satisfaction (1-5) – mean (standard deviation)	3.5 (1.5)	4 (0.98)	3.73 (1.3)	0.167
Failure Rate (percentage)	4.5%	4.5%	4.5%	1

**Figure 4:**
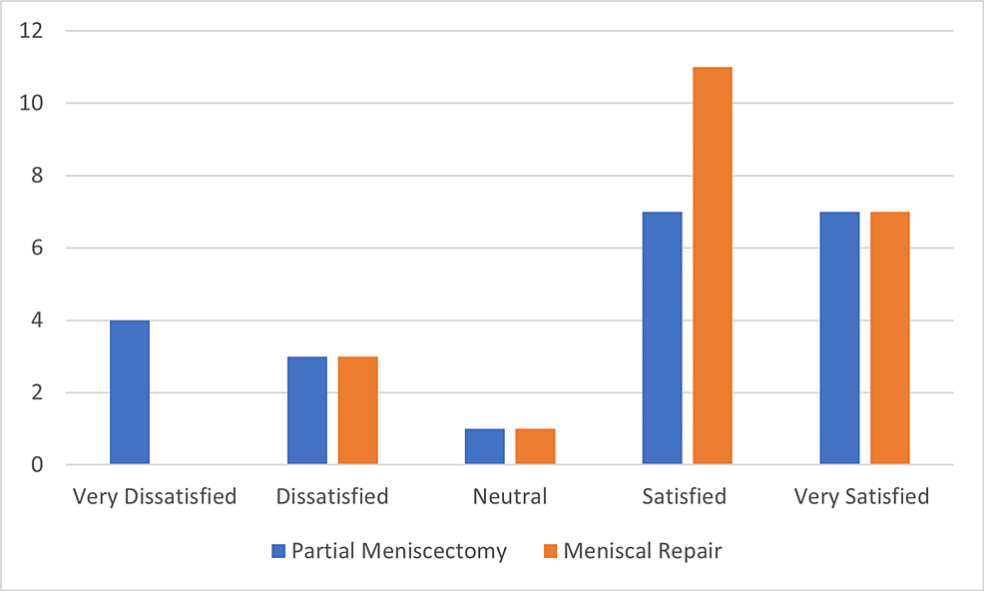
Distribution of the degree of patient satisfaction according to the surgical treatment (frequency)

With regards to the degree of patient satisfaction with the surgery, we found that the majority of patients were “Satisfied” or “Very Satisfied” (73%). Comparing the degree of satisfaction between both groups, we found no statistically significant difference between the two groups (p=0.167) (Table 2 and Figure 4).

**Figure 5:**
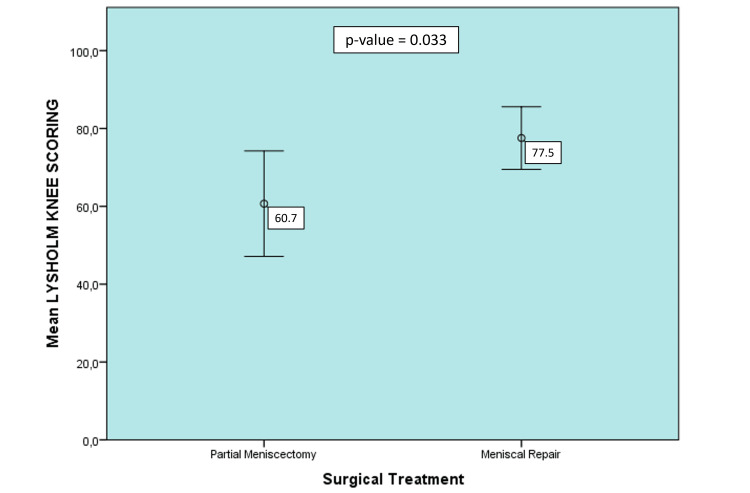
Functional Score according to the surgical treatment

Regarding the functional outcome, given by the Tegner Lysholm Knee Scoring Scale, on average, the group that underwent meniscal repair obtained a statistically superior score compared to the PM group (77.5 vs. 64.7; p-value 0.033) (Figure 5).

We observed a failure rate of 4.5% in each group. In the partial meniscectomy group, one patient needed additional surgery (total knee arthroplasty) due to failure to resolve the symptoms after the meniscectomy. Similarly, in the meniscal repair group, 1 patient also needed additional surgery (unicompartmental knee arthroplasty) for the same reason.

## Discussion

Meniscal injuries are classified based on their pattern and spatial orientation into vertical longitudinal (includes bucket handle), horizontal, radial, oblique, and complex injuries. The biomechanical implications of each type of tear correlates to the orientation of the collagen-fiber matrix of the meniscal microstructure and its disruption secondary to the tear [10].

Degenerative tears are frequently horizontal or complex tears, accompanied by osteoarthritic changes (chondral lesions of Outerbridge II and greater) [10,21-23]. This was observed in >85% of the cases, compared to 12% of knees that presented radial tears and 0% with vertical longitudinal tears, according to Mesiha et al. [16]. In addition, degenerative tears are classically seen in middle-aged (> 40 years old) or older people where the medial meniscus is the most frequently affected, and multiple tears are present in more than a third of patients [21].

Looking at the results of our study, we found a tear pattern prevalence similar to that described in the literature. In our study, patients over the age of 40 had a predominance of injuries in the medial meniscus located mainly on the body and posterior horn, and classified as horizontal cleavage or complex tears. All these characteristics are compatible with a degenerative tear pattern [10].

Age has been identified as a risk factor for meniscal injuries; in fact, population studies demonstrate that meniscal tears requiring treatment are 2-3 times more frequent in patients ≥40 years than in those under 40 years [4,5]. Age is also related to a decrease in intrinsic and perimeniscal cellularity [16]. In addition, blood supply to the menisci also changes with age. In a study conducted by Petersen and Tillmann [24], the authors evaluated 20 human menisci ranging in age from birth to 80 years old. They found that at birth, the whole meniscus was vascularized, and by the second year it was present as an avascular area on the inner circumference. By 20 years old, vascularization was only present in the peripheral third, which further regressed to a quarter at 50 years old, thus reducing healing potential [1,24]. This constituted the reason why age was classically described as a relative contraindication for meniscal repair with higher failure rates [1,25].

However, while meniscectomy leads to fewer reoperations, a successful meniscal repair results in improved long-term radiographic and clinical outcomes [10,22]. Recently, some studies have shown that even with an age superior to 40 years, a good outcome can be achieved with meniscal repair. One of the first authors to describe it was Barrett et al. in 1998 [12]. In their study with 37 patients (mean age of 44.2 years old), the early clinical success rate was 86.5% at 26.5 months, and only 5 patients had a recurrence of clinical symptoms [12]. With an overall clinical success rate of 86.5%, it demonstrated that with proper meniscal tear selection and surgical technique, meniscal healing rates obtained in younger patient populations could also be achieved in the older patient population [12]. 

The results of our study are consistent with these findings. We found that both surgical techniques (partial meniscectomy and meniscal repair) presented good short-term outcomes, and were effective in the treatment of meniscal tears in patients ≥40 years old, with a statistically significant decrease in the VAS score for pain when comparing pre and post-surgical periods. Despite the slight tendency for better outcomes in the MR group, no significant differences were found between both groups regarding pain relief and patient satisfaction with the surgery.

However, when studying the functional outcome, we found that the group that underwent meniscal repair obtained a significantly higher Tegner Lysholm Knee Score compared to the partial meniscectomy group. This was also consistent with the studies indicating that a healed meniscal repair produces an improved long-term functional outcome when compared with partial meniscectomy [10,26]. Moreover, meniscal repair is protective against the development and progression of arthritis, which relates to declining functional outcomes scores [27]. Despite these findings, we must also take into account that cartilage degenerative lesions may constitute a confounding factor that could influence functional outcomes. In our study, although the difference was not statistically significant, we observed a higher percentage of chondral lesions classified as grade 4 (Outerbridge Classification) in the partial meniscectomy group, and these, by themselves may be associated with a worse functional outcome.

Regarding the failure rate, our study denoted that the complications rate and the need for additional surgery were equal in both groups. In other words, in patients aged 40 or older, the meniscal repair did not present a higher failure rate than the meniscectomy. Recent literature supports these findings. In a study by Poland et al. (2019) with 56 patients over 40 years old, the authors concluded that the age of 40 years or older is not correlated with an increased risk of meniscal repair failure within 5 years of follow-up, although a shorter time to failure was noted [18]. Everhart et al. (2018) conducted a systematic review to evaluate published outcomes for reported failure rates following meniscus repair in patients > 40 years old. From the 225 articles found in the literature about meniscal repair in adults, only 11 presented outcomes for patients in this age group (148 patients). The results of this review demonstrated that the success rates for meniscal repair in this age group are comparable to those of younger patients. The failure rate among all patients ≥40 years was 10% and ranged 0-23% in individual studies with more than one patient in this age group. They found that successful repair was obtained up to age 70 years, and the failure rate in the oldest reported patients among studies with patients over age 60 years was 20%. The authors stated that age, as an independent factor, should not be considered a contraindication for meniscus repair [19]. Furthermore, a recent systematic review from Rothermel et al. [22] found no significant difference in failure rates between patients younger than 40 years versus older patients. However, this review did not look specifically at patients over 40 years, and analyzed a fairly small number of patients in this age group (n=28) [22].

We must highlight that these results should be considered taking into account our study's inherent limitations. This study had a small sample and this sample may not be representative of the overall population of patients ≥40 years old with meniscal injuries. We also note the potential heterogeneity of patients in both groups: they presented with different tear configurations in medial and the lateral meniscus, diverse locations with different proximity to vascularity, and some with concomitant ACL reconstructions. All these variables may work as potential confounders. Another confounder for repair success was that the repair technique may change according to the location of the meniscal injury, and the technique itself may influence the healing process. One more limitation that may provide a selection bias was the fact that the decision for meniscal repair versus partial meniscectomy was based on the surgeon’s experience and his assessment of the tear characteristics. Consequently, depending on the surgeon, the criteria for each surgical technique would be different, but can also mean that “better” tear patterns could be selected for meniscal repair, while “worse” tear patterns were selected for meniscectomy. This could produce a selection bias which could contribute to a better functional outcome in the MR group. However, these limitations do not completely invalidate the results, and some patients in this age group benefit from meniscal repair instead of meniscectomy. New studies, randomized and with a prospective design and greater cohorts, would allow us to prove and support our results.

## Conclusions

In patients aged 40 years or older that underwent a meniscal repair, we obtained a better functional outcome after 2 years compared to the group subjected to partial meniscectomy, and similar results in pain relief, patient satisfaction, and failure rate. In conclusion, age as an independent factor should not be considered a contraindication for meniscus repair. If technically possible, meniscal repair should always be performed, as it is associated with better functional outcomes, similar failure rates, and may be protective against the development and progression of arthritis.
